# Differentiating mouse embryonic stem cells express markers of human endometrium

**DOI:** 10.1186/s12958-017-0273-2

**Published:** 2017-07-17

**Authors:** P. Parasar, C. R. Sacha, N. Ng, E. R. McGuirk, S. Chinthala, P. Ozcan, J. Lindsey, S. Salas, M. R. Laufer, S. A. Missmer, R. M. Anchan

**Affiliations:** 1 Boston Center for Endometriosis, Boston Children’s and Brigham and Women’s Hospitals, 333 and 221 Longwood Avenue, Boston, MA 02115 USA; 2 Center for Infertility and Reproductive Surgery, Department of Obstetrics, Gynecology, and Reproductive Biology, Brigham and Women’s Hospital and Harvard Medical School, 75 Francis Street, Boston, MA 02115 USA; 3 Department of OB/GYN, University of Chicago, 5841 S. Maryland Ave, Chicago, IL 60637 USA; 4 Division of Gynecology, Department of Surgery, Boston Children’s Hospital, 300 Longwood Avenue, Boston, MA 02115 USA; 5 Division of Adolescent and Young Adult Medicine, Department of Medicine, Boston Children’s Hospital and Harvard Medical School, 300 Longwood Avenue, Boston, MA 02115 USA; 6 Department of Epidemiology, Harvard T. H. Chan School of Public Health, 677 Huntington Ave, Boston, MA 02115 USA

**Keywords:** Mouse embryonic stem cells, Endometrium, Differentiation, Animal model, Endometriosis

## Abstract

**Background:**

Modeling early endometrial differentiation is a crucial step towards understanding the divergent pathways between normal and ectopic endometrial development as seen in endometriosis.

**Methods:**

To investigate these pathways, mouse embryonic stem cells (mESCs) and embryoid bodies (EBs) were differentiated in standard EB medium (EBM). Immunofluorescence (IF) staining and reverse-transcription polymerase chain reaction (RT-PCR) were used to detect expression of human endometrial cell markers on differentiating cells, which were sorted into distinct populations using fluorescence-activated cell sorting (FACS).

**Results:**

A subpopulation (50%) of early differentiating mESCs expressed both glandular (CD9) and stromal (CD13) markers of human endometrium, suggestive of a novel endometrial precursor cell population. We further isolated a small population of endometrial mesenchymal stem cells, CD45−/CD146+/PDGFR-β+, from differentiating EBs, representing 0.7% of total cells. Finally, quantitative PCR demonstrated significantly amplified expression of transcription factors *Hoxa10* and *Foxa2* in CD13+ EBs isolated by FACS (*p* = 0.03).

**Conclusions:**

These findings demonstrate that mESCs have the capacity to express human endometrial cell markers and demonstrate potential differentiation pathways of endometrial precursor and mesenchymal stem cells, providing an in vitro system to model early endometrial tissue development. This model represents a key step in elucidating the mechanisms of ectopic endometrial tissue growth. Such a system could enable the development of strategies to prevent endometriosis and identify approaches for non-invasive monitoring of disease progression.

**Electronic supplementary material:**

The online version of this article (doi:10.1186/s12958-017-0273-2) contains supplementary material, which is available to authorized users.

## Background

Endometriosis is a chronic, hormone-dependent, inflammatory condition marked by growth of endometrial-like tissue at extra-uterine sites. Clinical symptoms include acyclic pelvic pain, dysmenorrhea, dyspareunia, dysuria, dyschezia, and infertility that can detrimentally impact a patient’s quality of life [1, 2]. Treatment and diagnosis of this disease is largely limited to surgical and hormonal approaches with a paucity of targeted treatments. In order to understand the pathophysiology of this disease, which is critical to developing better diagnostic and interventional tools, the early pathways of normal endometrial growth and development must be described.

Human uterine endometrium is an exceedingly dynamic tissue. Endometrial development and regrowth are supported by adult stem cells residing in the basalis region of the endometrium. These cells appear to possess tremendous regenerative potential, as demonstrated by their capacity to continually replenish the endometrial lining during the reproductive life of a woman [3]. During the menstrual cycle, these progenitor cells differentiate into *functionalis* glandular and stromal tissue. Although epithelial and mesenchymal stem cells have been identified in endometrial tissue, the disparate pathways that lead to normal versus ectopic endometrium remain unclear [4, 5]. Understanding the source of endometrial progenitor cell populations will further define the progression of endometrial pathology, in particular endometriosis, which has underlying genetic, hormonal, inflammatory, and immunological mechanisms that are not yet fully understood [6–9].

Embryonic stem cells are self-renewable cells with the capacity to differentiate into any tissue type, making them an invaluable tool to study mechanisms of pathogenesis through disease models [10]. The ability to produce endometrium from stem cells in vitro offers a way to investigate both normal and ectopic endometrial tissue development and identify endometrial progenitor cells. Prior research has demonstrated that neonatal mouse uterine mesenchyme (in the presence of endometrial growth factors Bone Morphogenetic Protein 4 (BMP4) and Activin A in serum free BPEL (Bovine Serum Albumin (BSA) Polyvinylalcohol Essential Lipids) medium) can induce human embryonic stem cells to differentiate in vivo towards mesendoderm, an intermediate stage of female reproductive tract epithelium development [11]. It has also been shown that endometrial-like cells can be generated from human embryonic stem cells co-cultured with endometrial stromal cells [12]. However, no studies have yet modeled endometrial growth utilizing mESCs, which are more available, easier to grow, require less time in culture, and are less expensive compared to human embryonic stem cells [13]. Most importantly for the study of human endometrium, there is a high degree of homology between the antigens expressed in human and mouse endometrium (Additional file 1: Table S1) [14].

Stem cell-derived endometrial precursor cells in culture may be identified by the expression of cell surface antigens found in human endometrial glandular and stromal cells: endometrial glandular epithelial marker CD9; stromal marker CD13; and co-expressed CD146 and platelet-derived growth factor receptor beta (PDGFR-β), specific for endometrial perivascular stromal cells and previously shown to be a source of human endometrial mesenchymal stem cells [15–17]. Furthermore, several transcription factors have been identified in early endometrial development, including *Hoxa10*, a transcriptional regulator that prior studies suggest could be involved in directing endometrial stromal cell differentiation and is key for normal implantation; [18–21] *Hand1*, which appears to be essential to trophoblast development; [22] and *Foxa2*, which is expressed in the glandular epithelium and contributes to implantation and fertility in mice [23].

In this study, we hypothesized that a mouse embryonic stem cell model could be designed to better understand early endometrial differentiation pathways in humans. We demonstrate differentiation of mESCs into a heterotopic cell culture system in which early co-expression of CD9 and CD13 was observed with IF staining and RT-PCR. We further assessed early endometrial differentiation pathways in EBs that illustrated distinct CD9, CD13, CD146+/PDGFR-β+, and *Hoxa10* expression. Additionally, using FACS, we isolated CD13+ cells from EBs with amplified levels of *Hoxa10*, *Foxa2*, and *Hand1*, as well as a subpopulation of CD146+/PDGFR-β + cells. Our results demonstrate a potential in vitro model of mESC-derived endometrial precursor cell development by elucidating early differentiation of cell marker expression and successfully isolating specific cell subpopulations with further regenerative potential.

## Methods

### Endometrium preparation

All mouse experiments were performed with the approval of the IUCAC (HMS IUCAC approval #05200). Endometrium from the uteri of six to seven-week old mice of strain 100,010 = B6C3F1/J was resected, snap-frozen in liquid nitrogen, and stored at minus 70 °C for cryosectioning and immunostaining procedures.

### Embryonic stem cell line and cell culture

A G4 mESC line (obtained from the laboratory of Dr. Andrew Nagy, Samuel Lunenfeld Institute, University of Toronto) was previously derived from a male blastocyst produced from the natural mating of a 129S6/SvEvTac female with a C57BL/6Ncr male [24–27]. G4 mESCs were maintained on Mitomycin C-inactivated mouse embryonic fibroblast feeder cells. mESCs were grown in mESC medium [Dulbecco’s Modified Eagle Medium (DMEM) (GIBCO, Carlsbad, CA), 10% ES-grade heat-inactivated fetal bovine serum (FBS) (Cellgro, Manassas, VA, USA), 100 U/ml ESCRO LIF (Millipore, Billerica, MA, USA), 2 mM L-Glutamine (GIBCO), and 0.2 mM 2-mercaptoethanol]. Cells were fed daily and split based on confluency.

For differentiation studies, mESCs were plated on gelatin-coated cell culture platforms for attachment and fed with DMEM with 10% serum.

### Generation of embryoid bodies

G4 mESCs were grown in three 10 cm cell culture plates until they were 70% confluent. In order to form an EB, i.e. three-dimensional aggregate of differentiating stem cells, mature mESC colonies were detached from the feeder layer with 0.05% trypsin-ethylenediaminetetraacetic acid (EDTA) (Gibco, ThermoFisher Scientific, Waltham, MA, USA) pre-warmed to 37 °C. Excess feeder cells were removed from the cell suspensions by serial plating on gelatin-coated plates, and the dissociated cells were transferred to low adhesion tissue culture plates coated with 2% poly-HEMA in ethanol. The cells were cultured in EBM [DMEM-F12, 15% Knock Out Serum Replacement (KOSR), 15% HI FBS, 1 mM L-glutamine, 0.1 mM 2-mercaptoethanol, and 1% Non-essential amino acids (NEAA, Invitrogen, Grand Island, NY), 1% antibiotic-antimycotic solution (Invitrogen), and 40 ng/ml human Beta fibroblast growth factor (β-FGF)] in suspension for a minimum of 14 days, during which they formed EBs.

The culture medium was changed every three days without disturbing the EBs for two weeks. Suspended EBs were transferred to gelatin-coated plates for attachment for three weeks for further differentiation in EBM. EBs were then examined by IF staining or collected for RNA isolation for quantitative and qualitative PCR analysis.

### Immunofluorescence staining

Prior to fixation, cells or tissue sections were briefly rinsed with phosphate buffered saline (PBS). Fixative (ice cold 4% Paraformaldehyde with 4% sucrose) was added to the specimen and cells were incubated for 15 min at room temperature (RT) with three subsequent rinses with PBS for five minutes each. Permeabilization was carried out by incubating the specimen with 1% Triton-X in PBS for 20 min at RT followed by rinsing with PBS three times for five minutes each. To block the non-specific binding sites, blocking buffer made of 1% bovine serum albumin (BSA) and 2% donkey serum in PBS was added to the specimen, which was then incubated for 30 min at RT. Primary antibodies CD9, CD13, CD146 and PDGFR-β diluted 100-fold in blocking buffer were added and the specimens were incubated for two hours at RT. Excess antibody was rinsed off with PBS three times and the corresponding Alexa Fluor-488 or Alexa Fluor-594 secondary antibodies (Invitrogen) (1:1000 in PBS) were added to the specimen and incubated for one hour in darkness. Following three PBS rinses, the nuclei were then counterstained with 4′, 6-diamidino-2-phenylindole (DAPI) diluted in PBS (1 μg/mL). The staining was visualized using dual color filter-fluorescence microscope (Zeiss, Pleasanton, CA, USA) with AxioVision analysis software. This protocol was used for mESCs and EBs.

Control mouse endometrium was stained for human endometrial markers CD9 (rat monoclonal Ab (mAb) CD9 PE, Ab82394, Abcam Inc., Cambridge, MA, USA), CD13 (rat mAb CD13 PE, Ab33490, Abcam Inc., Cambridge, MA, USA), CD45 (rat mAb CD45 PerCP, Ab82408, Abcam Inc., Cambridge, MA, USA), CD146 (mouse mAb CD146 FITC, Ab78451, Abcam Inc., Cambridge, MA, USA), and PDGFR-β (rabbit polyclonal Ab, Sc-432, Santa Cruz Biotechnologies, USA), as well as cytokeratin (Santa Cruz Biotechnology Inc., Dallas, TX, USA), vimentin (Sigma-Aldrich, St. Louis, MO, USA), Rabbit (Rb) polyclonal E-cadherin (Abcam Inc., Cambridge, MA, USA), Rb polyclonal estrogen receptors A (ER-A, Abcam Inc., USA) and B (ER-B, Santa Cruz Biotechnologies, Dallas, TX, USA), progesterone receptors A and B (PR-A and PR-B, Abcam Inc., Cambridge, MA, USA), and HOXA10 (goat polyclonal Ab, Sc-17,159, Santa Cruz Biotechnologies, Dallas, TX, USA; Additional file 1: Table S1).

Mouse ESCs differentiating in DMEM with 10% serum for three weeks were stained for CD9 and CD13; cell counts in seven different views were performed and the mean percentage of positively-staining cells was calculated for qualitative comparison. EBs were stained for CD9, CD13, CD146 and PDGFR-β, and HOXa10 using the protocol detailed above. Three independent technicians then performed cell counts in seven different views, from which a mean percentage of positive cells was calculated for each cell marker. Staining was performed twice on the mESC and EB samples.

### RNA isolation and reverse transcription

Total RNA was isolated from mouse uterine samples, G4 mESCs, and EBs using a QIAGEN kit (QIAGEN Inc., Germantown, MD, USA) according to the manufacturer’s protocol. RNA yield was quantified by UV spectroscopy, and 1-2 μg of RNA were treated with RNAse free DNAse and reverse transcribed using qScript reverse transcriptase with random primers (Quanta Biosciences, Gaithersburg, MD, USA) for RT-PCR.

### RT-PCR

RT-PCR analyses were performed on EBs at Week 1, 2 and 3 of differentiation using primers for endometrial cell markers. RT was performed using the qScript cDNA Synthesis Kit (Quanta Biosciences, Gaithersburg, MD, USA). The RT product was used in PCR with the GoTaq Core System I (Promega, Fitchburg, WI, USA) for 35 amplification cycles at a 57–60°Celsius (C) annealing temperature. A 25 μl reaction contained 2 ul MgCl_2_ (1 mM), 5 ul 5X Green Flexi Buffer (1X), 0.5 ul PCR nucleotide mix (0.1uM), 1 ul each of forward and reverse primers (0.1uM), 0.125 ul of GoTaq polymerase (0.625 units), 2 ul of cDNA, and nuclease free water to make final volume 25 ul. Reactions were placed in thermal cycler and amplified at 95 °C for 2 min, 35 cycles of initial denaturation at 94 °C for 30 s, annealing temperature of 55 °C for 30 s, extension of 72 °C for 30 s, final extension of 72 °C for 10 min, and hold at 4 °C. The PCR products were analyzed by agarose gel electrophoresis.

### Flow Cytometry

EBs differentiated in EB medium for three weeks were dissociated using 0.05% trypsin. Cells were enumerated using a hemocytometer. One million cells were used for flow cytometry staining. Cells were washed twice with flow buffer (FB) (DMEM with 1% BSA) by centrifuging the cells at 800 xg for 3 min. Primary antibodies (conjugated CD45, CD9, CD13, CD146 and unconjugated PDGFR-β) were diluted with 15 μg/ml of FB, added to the cells, and the mixture was incubated for one hour on ice in darkness. Following two washes with FB, the PDGFR-β sample was added with secondary Alexa fluor-647-conjugated secondary antibody (Invitrogen) and incubated for 30 min on ice in darkness with two washes afterwards with FB. Samples were re-suspended in FB at a concentration of one million cells per 500 μl of buffer. Prior to analysis, DAPI was added to the cells at a concentration of 3uM.

CD13+ and CD146+/PDGFR-β + cells were sorted using a BD FACS Aria IIu sorter (BD Biosciences, San Jose, CA, USA) at the Dana Farber Flow Cytometry Lab. Two flow cytometry runs of cultured EBs were done, with sorting performed if cell count was adequate. The sorts with the highest cell counts were analyzed. From the total cell population, viable cells were negatively selected with DAPI, and CD45+ cells were excluded to avoid the differentiating leukocyte population and bone marrow-derived epithelial and stromal cells. Debris cells were excluded by gating based on forward scatter (FSC) and side scatter (SSC). CD13+ and CD146+/PDGFR-β + cells were sorted from the viable CD45– subpopulation of differentiated cells. Flow cytometry scatter data was gated and imported into plots using FlowJo software. The CD13+ cell fractions were set aside for RNA extraction for quantitative RT-PCR.

### Quantitative RT-PCR

Quantitative RT-PCR was performed to evaluate the relative expression of *Hoxa10*, *ER-A* and *-B*, and *PR-A* and *-B* in EBs, CD13+ cells, and CD13– cells. Mouse beta-actin was used as an endogenous control gene. Primer sequences were selected from the Harvard PrimerBank (http://pga.mgh.harvard.edu/primerbank/index.html) (Additional file 1: Table S2).

The reverse transcription was carried out using a Bio-Rad PCR machine (Bio-Rad, Hercules, CA, USA) and Platinum *Pfx Taq* polymerase (ThermoFisher Scientific, Waltham, MA, USA). Cycle parameters were initial denaturation at 94 °C for 2 min, 30 cycles of initial denaturation at 94 °C for 30 s, annealing temperature of 55 °C for 30 s, extension of 68 °C for 30 s, final extension of 68 °C for 10 min, and hold at 4 °C. Quantitative RT-PCR was performed using an iCycler iQTM Real-Time PCR Detection System (Bio-Rad, Hercules, CA, USA). Assays were carried out in triplicate with three wells for each sample in optically clear thin-walled PCR plate sealed with optical sealing tape (Eppendorf, Brinkmann, Westbury, NY, USA). Reactions (20 μl) comprised *Power* SYBR® Master Mix (ThermoFisher Scientific, Waltham, MA, USA) plus forward and reverse primers at 500 nM concentration, and 1 μl of undiluted cDNA. A standard PCR protocol was used for all primers: 10 min at 95 °C for AmpliTaq Gold DNA Polymerase (ThermoFisher Scientific, Waltham, MA, USA) activation; 40 cycles of 15 s at 95 °C, 30 s at 60 °C, and 30 s at 70 °C with data collection enabled; 1 min at 95 °C; 1 min at 55 °C; and an 80 cycle melt curve analysis with temperature increasing by 0.5 °C per cycle.

The data was obtained with iQ5 Optical System Software Version 2.1 (Bio-Rad, Hercules, CA, USA) using the cycle threshold (Ct) method. The raw quantity of each run was standardized relative to the beta actin control, and the means and standard deviations were calculated using JMP Software Version 13 (SAS, Cary, NC, USA). Mann-Whitney U and Kruskal-Wallis nonparametric tests were used to compare the gene expression distribution in the EB, CD13+, and CD13- populations.

### Microarray mRNA expression analysis

Microarrays were run at the Boston Children’s Hospital Microarray core facility using the Illumina WG-6 Version 2.0 Kit (Illumina, San Diego, CA, USA). cDNA was amplified from RNA extracted from EBs and run in triplicate. Our analyses compared differentiating mouse EBs with undifferentiated mESCs. Data analysis was performed with the R statistical environment (http://www.r-project.org), and the microarray data were processed using the *lumi* package for background subtraction, log2 transformation, and quantile normalization, as described previously [28]. To filter out unresponsive probes, we removed probes that had detection *P*-values <0.01 (as determined by Illumina’s BeadStudio software) in less than or equal to one sample across all samples (3 samples in total). The remaining 25,294 probes were used for analysis. The final dataset contained 18,029 genes. Hierarchal clustering of genome-wide expression as well as genes involved in endometrial development and steroidogenesis are demonstrated using heat maps. Principal component analysis (PCA) was performed on the gene expression dataset using R’s *prcomp* function. Differential expression analysis was performed using limma. Next, the common differentially regulated genes (DRGs) and relevant gene regulatory networks (GRNs) were evaluated. Differential gene expression specifically relevant to endometrium and endometriosis development was further analyzed using Ingenuity Pathways Analysis (IPA) software (Ingenuity® Systems, Redwood City, CA, USA).

## Results

### Mouse endometrium expresses human endometrial cell markers

**Fig. 1:**
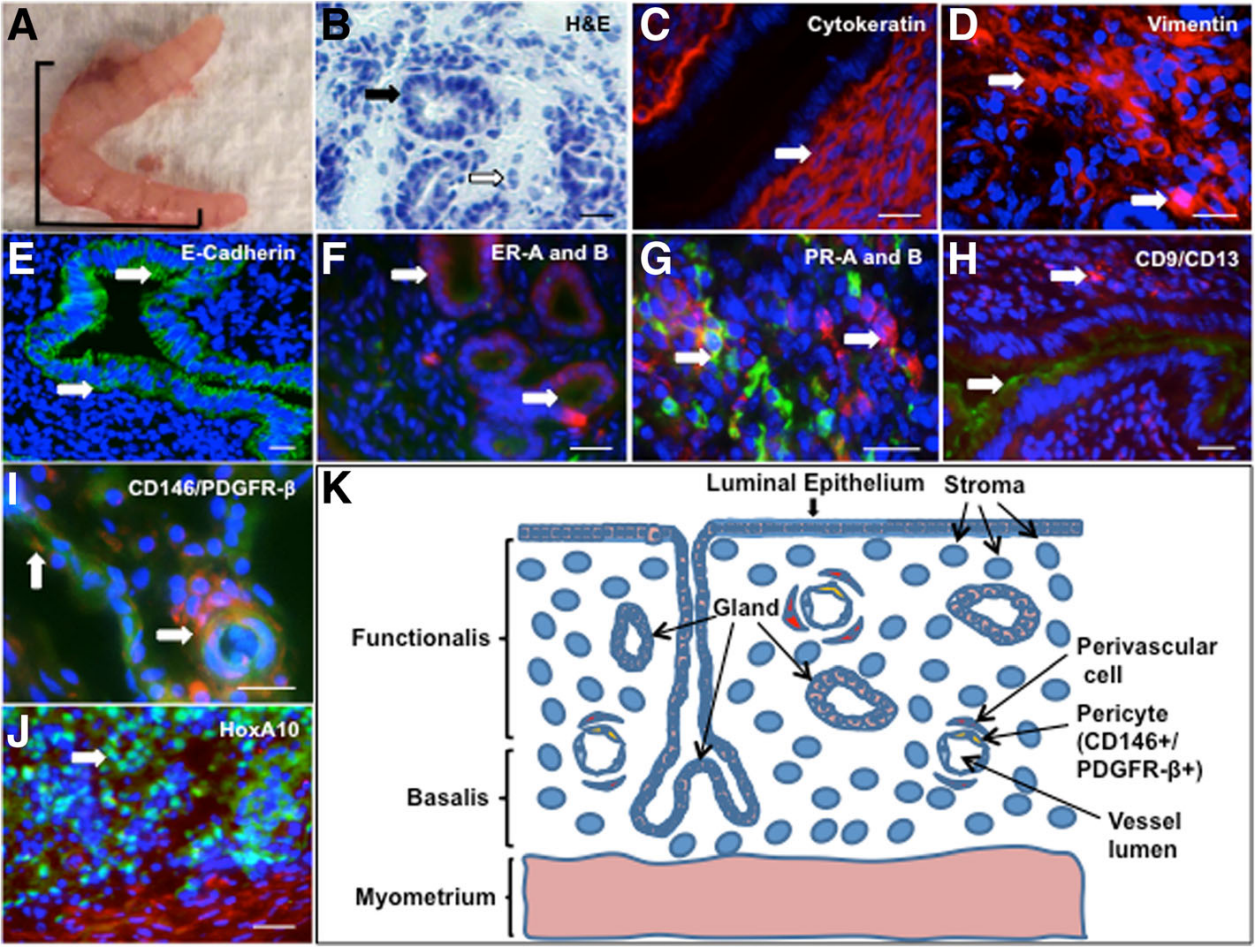
Mouse endometrium expresses markers of human endometrium. Gross murine uterus is shown in (**a**) (1 cm scale) followed by H&E staining of mouse endometrium (**b**; black arrow indicates glandular architecture and black arrow indicates stroma). Immunofluorescence staining for extracellular matrix markers cytokeratin, vimentin, and E-Cadherin is shown in (**c**, **d** and **e**), respectively. Expression of ER-A (*green*) and ER-B (red) (**f**), PR-A (*green*) and PR-B (*red*) (**g**), CD9 (*green*) and CD13 (*red*) (**h**), PDGFR-β/CD146 cell markers (**i**), and HOXa10 (*green*) (**j**) is shown in mouse endometrium. A schematic of the distribution of cell populations of interest is shown in (**k**). White arrows indicate positive expression; scale bars at bottom right of panels B-J correspond to 20 μm

Mouse endometrium, shown grossly and by H&E staining in Fig. 1a and b, is similar in its glandular and stromal composition to human endometrium (Fig. 1j; Additional file 1: Table S1). Mouse endometrium expresses non-specific markers of human endometrium, such as cytokeratin, vimentin, and E-cadherin (Fig. 1c, d, and e, respectively), ER-A and ER-B (Fig. 1f), and PR-A and PR-B (Fig. 1g). CD9 was expressed on the epithelium of the glands and CD13 on stromal cells in mouse endometrium (Fig. 1h). Mouse endometrial perivascular stromal cells co-expressed PDGFR-β and CD146 (Fig. 1i.) Finally, mouse uterine tissues expressed HOXa10 (Fig. 1j). A schematic of stromal and glandular cells is shown in Fig. 1k.

### mESCs differentiate into putative stem cell populations that co-express endometrial markers CD9 and CD13

**Fig. 2:**
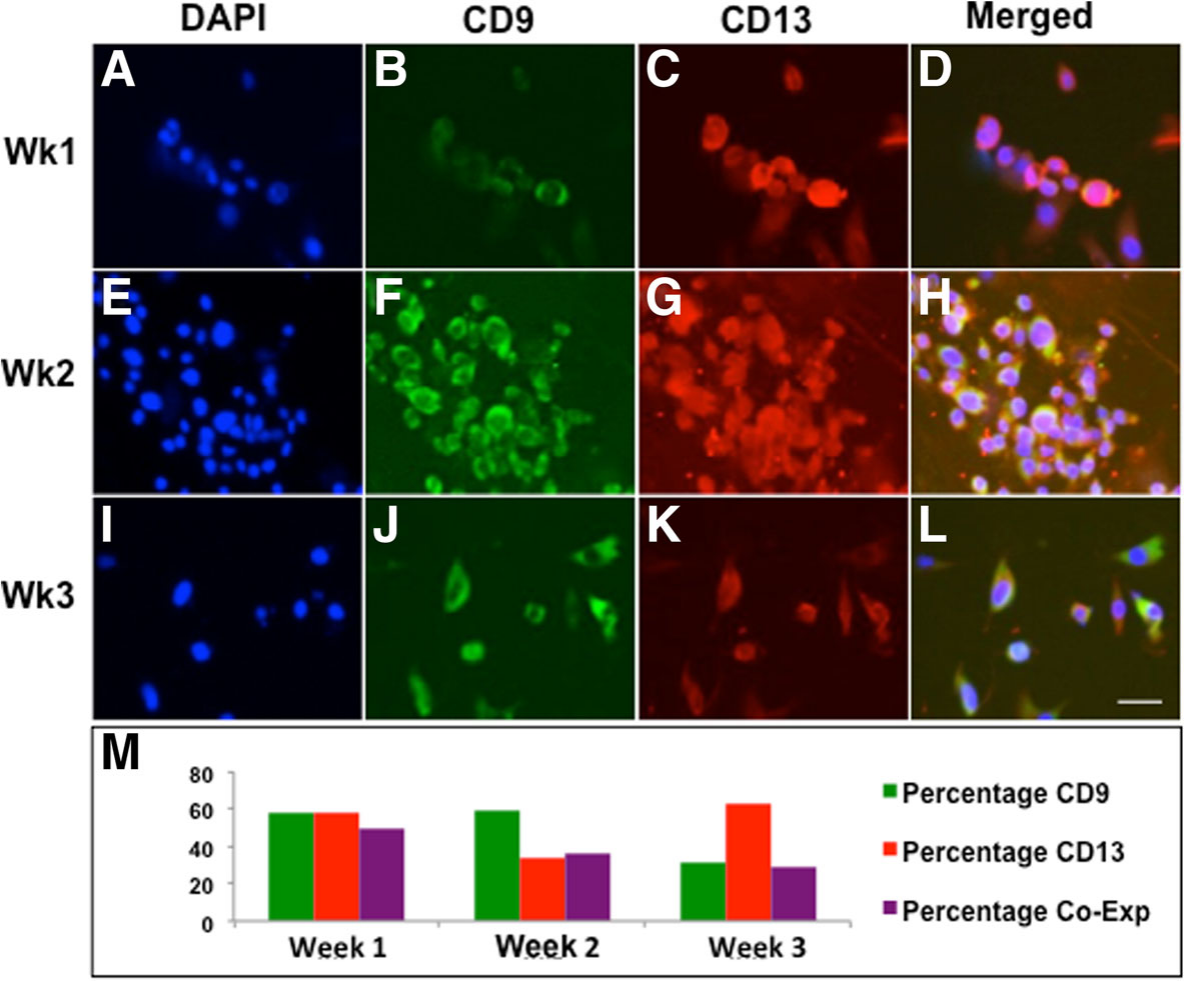
mESCs differentiate to express endometrial precursor cell markers CD9 and CD13 by three weeks in standard culture. By one, two, and three weeks in culture, mESCs display both human endometrial epithelial CD9 (**b**, **f**, **j**) and stromal CD13 cell markers (**c**, **g**, **k**). Co-expression is illustrated in (**d**, **h**, and **l**). As shown in (**m**), colonies of mESCs showed progressive differentiation of CD9 immunoreactive cells over a three-week period (58%, 60%, and 31% in weeks one, two, and three, respectively), while populations of differentiated CD13 cells increased over this same duration (57%, 34% and 62% in weeks one, two, and three, respectively). Bottom right scale bar in panel (**l**) indicates 20 μm for all panels

mESCs differentiate for three weeks in DMEM with 10% serum demonstrated evolving co-expression of CD9 and CD13 (Fig. 2a-l.) The total cell count peaked at week two and decreased by week three. While the proportion of CD9 immunoreactive cells decreased from week two to three (58% in week one, 60% in week two, and 31% in week 3, Fig. 2m), the proportion of differentiated CD13 cells was highest in week three (57%, 34% and 62% in weeks one, two, and three, respectively, Fig. 2m). The proportion of CD9+/CD13+ cells decreased over three weeks (50%, 35%, and 29%, respectively, Fig. 2m).

### Differentiating EBs express markers of endometrial glands, stroma and mesenchymal stem cells, confirmed by RT-PCR

**Fig. 3:**
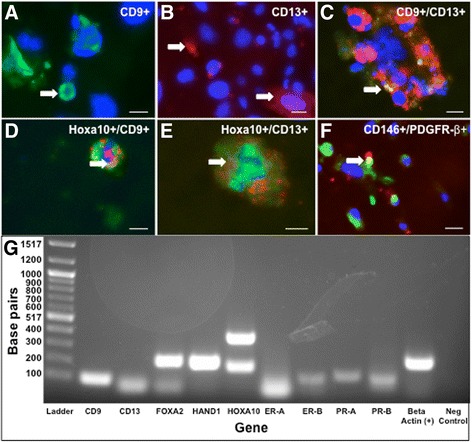
Mouse EBs express human endometrial markers, confirmed by PCR. EBs express human endometrium glandular cell marker CD9 (*green*, **a**), stromal marker CD13 (*red*, **b**). Minimal CD9/CD13 co-expression is observed at this point (**c**). HOXa10 co-expression (green) with CD9 and CD13 are shown in (**d** and **e**), respectively. Mesenchymal stem cell markers CD146/PDGFR-β are shown in (**f**) (*yellow*). White arrows indicate positive expression. Scale bars indicate 20 μm. RT-PCR confirmed expression of CD9 and CD13 as well as *Foxa2, Hand1, Hoxa10*, *ER-A* and *-B*, and *PR-A* and *-B* (**g**; ladder in left column, positive and negative controls shown in far right columns)

mESCs cultured on gelatin in EB media formed EBs that maintained CD9 and CD13 expression, observed by immunostaining (Fig. 3a-b). A small level of CD9/CD13 co-expression was also seen in EBs (Fig. 3c). HOXa10 co-expression with CD9 and CD13-specific cell populations and CD146/PDGFR-β co-expression was also noted (Fig. 3d-f). Average expression of the endometrial cell markers in EBs was 36% CD9+, 14% CD13+, 72.3% HOXa10, and 11% CD146+/PDGFR-β + of total cells. The range for CD9+, CD13+, HOXa10, and CD146+/PDGFR-β + cells were 1 to 148, 0 to 75, 7 to 89, and 0 to 22, respectively. RT-PCR of cultured EBs at Weeks 1, 2, and 3 confirmed expression of the endometrial cell markers (Fig. 3g, Additional file 2: Figure S1).

### FACS isolates CD13+ and CD146+/PDGFR-β + cells from differentiating EBs

**Fig. 4:**
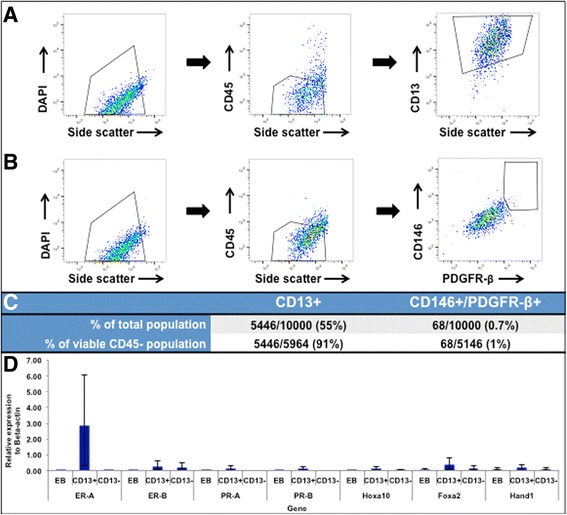
FACS sorting of CD13+ and CD146+/PDGFR-β + cells from mouse EBs. Total EB cells gated as DAPI+/CD45−/CD13+ (**a**) and DAPI+/CD45−/CD146+/PDGFR-β + (**b**) are shown. Graph legends indicate percent of parent population. **c** shows the proportion of CD13+ and CD146+/PDGFR-β + cells isolated from both the total cell population and viable CD45- population in EB medium by flow cytometry. Quantitative RT-PCR revealed significantly up-regulated expression of *Hoxa10*, *Foxa2*, *ER-A, PR-A* and *PR-B*, in a CD13+ (stromal) subpopulation (**d**). The x-axis represents the gene and the y-axis represents the relative fold change to the Beta-actin gene control. Standard deviations of means are shown with brackets

EBs were sorted with flow cytometry into stromal and glandular sub-components, though the CD9-specific cell counts were too low to be quantified. In the other sorted fractions, 55% CD13+ and 0.7% CD146+/PDGFR-β + cells were obtained from the total cell population, and 91% and 1% of the viable CD45– population were CD13+ and CD146+/PDGFR-β+, respectively (Fig. 4a-c Additional file 3: Figure S2).

### Quantitative RT-PCR illustrates selective increased endometrial cell marker expression in CD13+ cell population

Expression of *ER-A*, *ER-B*, *PR-A*, *PR-B*, *Hoxa10*, *Foxa2*, and *Hand1* were examined in EBs as well as the CD13+ and CD13- sorted cell populations to confirm isolation of precursor endometrial stromal cells. Relative expression levels are shown in Fig. 4d. *ER-A* expression in the CD13+ cells was highest compared to the other genes of interest (2.87; *p* = 0.004). *PR-A* and *PR-B* had greater expression in the CD13+ cells than in EBs (*PR-A:* 0.15 vs. 0.001 in EBs, *p* = 0.0495; *PR-B:* 0.12 vs. 0.001 in EBs, *p* = 0.0495). *Hoxa10* was expressed at a higher level in the CD13+ population than in the EBs or CD13- cells (0.11 vs. 0.001 in EBs and 0.03 in CD13- fraction; *p* = 0.03), as was Foxa2 (0.38 vs. 0.06 in EBs and 0.12 in CD13- fraction; *p* = 0.03). There was a trend towards greater expression of *ER-B* (*p* = 0.05) and *Hand1* in the CD13+ fraction that failed to reach statistical significance (*p* = 0.07). *PR-A* and *PR-B* expression was not detected in the CD13- cell fraction. *ER-B, Hoxa10, Foxa2, and Hand1* were expressed at similar low levels in the CD13- cell population (*p* = 0.06).

### Gene analysis by microarray demonstrates early differentiation of endometrial regulatory pathways in EBs

**Fig. 5:**
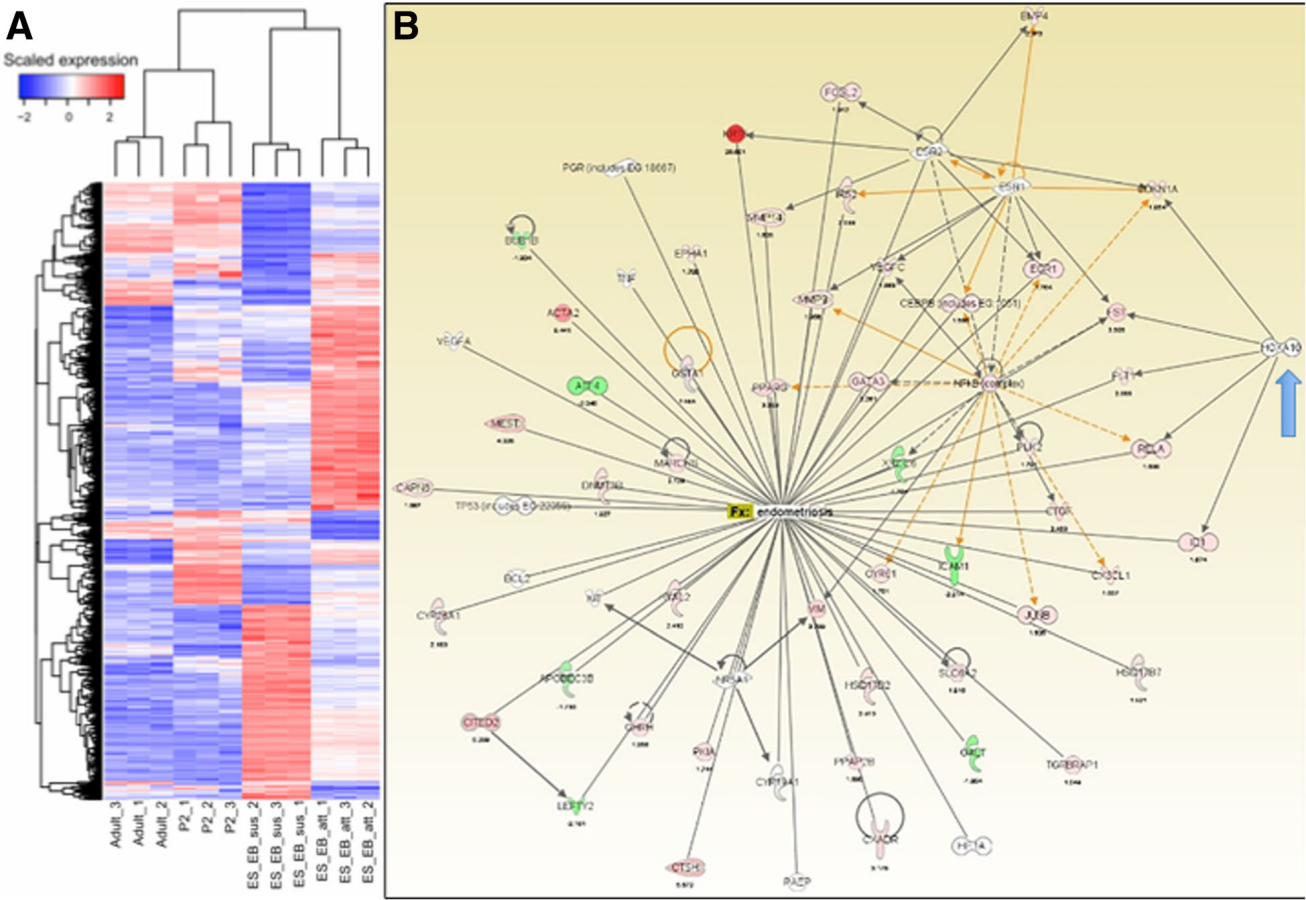
Microarray analysis of gene expression in EBs. A heat map (**a**) and IPA gene network (**b**) reveal the array of genes specific to endometrial and endometriosis development expressed in differentiating EBs. Hoxa10 is highlighted with the blue arrow on the right side of the image

Microarray analysis of differentiating EBs revealed emerging gene regulatory pathways related to the development of endometrium and endometriosis as shown in the heat map and IPA network of genes (Fig. 5a and b). The IPA network illustrates the presence of *ER-A, ER-B, PR-A, PR-B*, nuclear factor kappa beta (*NFκ*-β), matrix metalloproteinases (*MMP3*), vascular endothelial growth factor (*VEGF*), and *Hoxa10* genes in differentiating EBs.

## Discussion

Endometriosis affects nearly 10% of reproductive age women and can be debilitating. However, the absence of unique antigenic markers of ectopic endometrial lesions has severely limited therapeutic discovery for this disease. Current treatment is limited to surgical destruction and/or excision of lesions followed by hormonal therapy to delay symptom recurrence. Our understanding of the molecular mechanisms that underlie onset and progression of this disease remains incomplete. Therefore, embryonic and pluripotent stem cells provide us with a unique structure to develop an in vitro model for endometriosis and to better understand its underlying molecular mechanisms.

Endometrial stem cells have regenerative potential and are reportedly able to differentiate into chondrocytes, adipocytes, and osteoblasts [3, 29]. Once disseminated through retrograde menstruation, endometrial stem cells may allow endometrial cells to migrate and grow ectopically, developing into endometriosis. Currently, the full spectrum of abnormalities in ectopic endometriosis tissue and whether the immune system fails to recognize endometriotic implants as abnormal are active areas of research. It is also possible that normal endometrial tissue contributes to the pathology of endometriosis by acquiring aberrant genetic features.

In order to evaluate early patterns of endometrial cell differentiation from the embryonic stem cell stage, we first illustrated the expression of human endometrial cell markers in normal mouse endometrium to validate the use of a mouse model. We then studied mESC expression of CD9 and CD13, which are strongly expressed on endometrial glandular and stromal cell surfaces, respectively, in both normal and ectopic endometrium in humans [30]. Interestingly, differentiating mESCs in two-dimensional colonies appeared to generate a population of cells that were both CD9 and CD13 immunoreactive (Fig. 2). Since these antigens are typically associated with distinct human glandular and stromal endometrial cells, and with continued differentiation the co-expressing populations segregate into either CD9+ or CD13+ cell types, we hypothesize that this differentiation pathway may indeed represent a transient temporal differentiation state of primitive stem cells that subsequently commits to either a CD9-specific or CD13-specific histologic cell fate.

When differentiation was enhanced with three-dimensional aggregates (EBs), we were able to distinguish two separate immunogenic populations of cells. We noted emergence of distinct CD9+ (36%) and CD13+ cells (14%) (Fig. 3a-b), with minimal residual CD9/CD13 co-expression (Fig. 3c) and widespread expression of HOXa10 (72.3%) in EBs. Given that CD9 and CD13 are not unique to human endometrium—for example, CD13, is also expressed in the proximal tubules of the kidney [31]—we evaluated additional factors involved in endometrial growth and receptivity to further define early development. In particular, we confirmed expression of progesterone and estrogen receptors, *Hoxa10*, *Hand1,* and *Foxa2* in EBs at the early stages of endometrial development by RT-PCR (Fig. 3g, Additional file 2: Figure S1). We were then able to exploit the emergence of separate CD9+ and CD13+ cell populations to progressively enrich our cultures for endometrium-related precursor cells using FACS sorting in multiple stages (Fig. 4a-c). An IPA network of differentiating EBs confirmed that genes related to endometrial and endometriosis development, including *Hoxa10*, are expressed in EBs (Fig. 5a and b). Therefore, mESCs provide an opportunity to develop a valid animal model for early normal endometrium compared to ectopic endometrial development, which may help identify specific markers of endometriosis.

The CD13+ cell population demonstrated amplified expression of *ER-A*, *PR-A*, and *PR-B*, representing early localization of receptor development, particularly ER-A, with an associated statistically significant up-regulation in *Hoxa10* and *Foxa2* gene expression (*p* = 0.03, Fig. 4d). The CD13– cell population, which includes CD9+ cells, was notable for similar low-level expression of *ER-B, Foxa2, Hoxa10, and Hand1. ER-B* and *Foxa2* are both expressed in epithelial cells expected to be in the epithelial fraction [32–34]. Endometrial expression of *Hoxa10*, which plays a key role in implantation, has been shown to be reduced in ectopic endometrium and as well as in the eutopic endometrium of women with infertility [35, 36]. *Hoxa10* expression is notably highly conserved in the female reproductive tract of both mice and humans, making this gene a potential target in animal modeling of endometriosis development [37]. *Foxa2* appears to play a role in implantation, as well as adenogenesis (formation of uterine glands), which is critical for normal endometrial development [23]. *Hand1* was expressed similarly in the CD13+ and CD13– cell fractions. The Hand1 protein has been isolated in endometrial cancer samples and has been expressed preferentially in mESCs cultured with BMP4, allowing for trophoblast differentiation from mESCs, a common pathway with humans [22, 23, 38, 39]. The expression of these markers in EBs and precursor stromal cells supports their essential role in normal endometrial differentiation. We hypothesize that given the pseudomalignant behavior of endometriotic lesions, alterations in *Hand1* expression at an early stage of development may contribute to ectopic endometrial development. Future studies in this lab will further explore the role of these transcription factors in ectopic endometrial growth. The early expression of these markers in endometrial stromal precursor cells isolated from EBs will enable us to establish an in vitro co-culture system to explore the molecular mechanisms of endometriotic tissue development using mouse embryonic stem cells.

A mesenchymal stem cell population (CD146+/PDGFR-β+), previously isolated from human endometrial perivascular stroma, is a potential source of adult stem cells in endometrium, contributing to stromal vascular regrowth during menses [4, 14, 15, 40]. We successfully identified and isolated a small population of CD146+/PDGFR-β + cells from differentiating mESC-derived EBs. The presence of mesenchymal stem cell markers in differentiating EBs suggests that such cells could play an important role in the early human endometrial development and may therefore contribute to the proliferation of ectopic endometrium. The expression and differentiation of human endometrial progenitor cell markers in mESCs lay the foundation to isolate and enrich populations of early endometrial cells that have differentiated from embryonic stem cells. However, the complete role of mesenchymal stem cells in early endometrial development and the pathways that commit stem cells to endometrial precursor cells require further exploration.

## Conclusions

To our knowledge, this study is a novel display of human endometrial cell markers in early differentiating mESCs, with co-expression of endometrial cell markers CD9 and CD13 possibly signifying a unique, primordial endometrial multipotent precursor cell population in a mouse model. The presence of human endometrial and mesenchymal stem cell surface markers on mouse endometrium, as well as the differentiation of mESCs into a heterogeneous endometrial precursor cell culture system with expression of specific key markers of human endometrium, demonstrate the potential use of mESCs for modeling early normal and ectopic endometrial development in vitro. This system would allow for the future study of the key molecular pathways of endometriosis development and progression. While sensitive and specific biomarkers of endometriosis are yet to be determined, we aim to use this model to identify and examine crucial transcriptional markers implicated in disease progression. Identification of unique biomarkers would enable utilization of innovative techniques such as Clustered Regularly-Interspaced Short Palindromic Repeats (CRISPR) to inhibit target genes and thus arrest the development of endometrium and endometriosis. Employing cost-effective, efficient, and easily scalable mouse embryonic stem cells is an essential step towards using induced pluripotent stem cells derived from patients for personalized therapy in the medical management of endometriosis.

## Additional files

Additional file 1: Table S1. Markers for human endometrium. **Table S2.** Primer sequences used for quantitative real-time expression analysis. (DOCX 19 kb)

Additional file 2: Figure S1. Gene expression in differentiating EBs in Week 1 and Week 2. RT-PCR demonstrates expression of CD9, CD13, *Foxa2, Hand1, Hoxa10*, *ER-A* and *-B*, and *PR-A* and *-B* in Week 1 (A) and 2 (B) of EB differentiation. The ladder is indicated on the left and positive and negative controls are shown in far right columns. (TIFF 1521 kb)

Additional file 3: Figure S2. Schema of complete gating of FACS-sorted CD13+ cells in EB media. Total cells were first gated to exclude debris with forward and side scatter plots (A-C). Next, viable cells were negatively selected with DAPI (D). CD45- cells were selected (E), followed by CD13+ cells (F). Graph legends indicate percent of parent population. (TIFF 1521 kb)

## Abbreviations

BMP4: Bone Morphogenetic Protein 4
BPEL: Bovine serum albumin polyvinylalcohol essential lipids
BSA: Bovine serum albumin
CD: Cluster of differentiation antigen
CRISPR: Clustered regularly-interspaced short palindromic repeats
DAPI: 4′, 6-diamidino-2-phenylindole
DMEM: Dulbecco’s Modified Eagle Medium
EBM: Embryoid body medium
EBs: Embryoid bodies
EDTA: Ethylenediaminetetraacetic acid
ER: Estrogen receptor
ESCs: Embryonic stem cells
FACS: Fluorescence-activated cell sorting
FB: Flow buffer
FBS: Fetal bovine serum
FSC: Forward scatter
IF: Immunofluorescence staining
IPA: Ingenuity Pathways Analysis
KOSR: Knock Out Serum Replacement
mESCs: Mouse embryonic stem cells
MMP3: Matrix metalloproteinases
NFκ-β: Nuclear factor kappa beta
PBS: Phosphate buffered saline
PCA: Principal component analysis
PDGFR-β: Platelet derived growth factor beta
PR: Progesterone receptor
Rb: Rabbit
RT: Room temperature
RT-PCR: Reverse-transcription polymerase chain reaction
SSC: Side scatter
VEGF: Vascular endothelial growth factor
β-FGF: Beta fibroblast growth factor
