# Supplementary material for: Differentiating mouse embryonic stem cells express markers of human endometrium
Source: Reprod Biol Endocrinol. 2017 Jul 17;15:52. doi: 10.1186/s12958-017-0273-2 (PMC5514487; doi:10.1186/s12958-017-0273-2)
Supplement: Supplementary file 1 — Markers for human endometrium. Table S2. Primer sequences used for quantitative real-time expression analysis. (DOCX 19 kb) [file 12958_2017_273_MOESM1_ESM.docx]

**SUPPLEMENTAL TABLES**

**Table 1: Markers for human endometrium**

| **Markers** | **PCR product size (base pair)** | **Endometrial cells expressing the receptors** | **Identity percentage with human markers (%) (with accession numbers)** |
| --- | --- | --- | --- |
| CD9 | 103 | Glandular epithelial cells | 89 (Acc#NP_001760 and NP_031683.1) |
| CD13 | 152 | Stromal cells | 76 (NP_032512 and BAD93155.1) |
| CD146^+^/PDGFR-B^+^ | * | Endometrial mesenchymal stem cells | CD146 (76%),  (AAH26985 and NP_006491.2) PDGFR-B (85%)  (NP_001139740 and AAH32224.1) |
| Estrogen receptor alpha | 128 | Epithelial, stromal and vascular cells | 89 (AAI28575.1 and ACF17956.1) |
| Estrogen receptor beta | 129 | Epithelial, stromal and vascular cells | 86 (NP_997590.1 and NP_001428.1) |
| Progesterone receptor | A (135)  B (142) | Epithelial and stromal cells | 80 (NP_032855.2 and AAD01587.1) |
| E-Cadherin | * | Glandular cells | 81 (NP_033994.1 and CAA79356.1) |
| Cytokeratin | * | Endometrial epithelial cells | 87 AAA37551.1 and CAA67203.1) |
| Vimentin | * | Stromal cells | 97 (NP_035831.2 and NP_003371.2) |
| Hoxa10 | 196 | Epithelial and stromal cells | 86 (NP_032289.2 and [NP_061824.3](https://www.ncbi.nlm.nih.gov/protein/182765442?report=genbank&log$=prottop&blast_rank=53&RID=MX46K7BC015)) |
| Foxa2 | 222 | Epithelial and stromal cells | 97 (AAX90601.1 and [NP_710141.1](https://www.ncbi.nlm.nih.gov/protein/24497504?report=genbank&log$=prottop&blast_rank=22&RID=MX3ZN2PJ015)) |
| Hand1 | 215 | Epithelial and stromal cells | 92 (EDL07780.1 and [NP_004812.1](https://www.ncbi.nlm.nih.gov/protein/4758506?report=genbank&log$=prottop&blast_rank=60&RID=MX4AXC3X015)) |

*Not used in PCR experiments

**Table 2. Primer sequences used for quantitative real-time expression analysis**

| **Gene** | **Forward** | **Reverse** |
| --- | --- | --- |
| Beta-actin | AGAGCTACGAGCTGCCTGAC | AGCACTGTGTTGGCGTACAG |
| Mus musculus CD9 | ATGCCGGTCAAAGGAGGTAG | GCCATAGTCCAATAGCAAGCA |
| Mus musculus CD13 | AGGGGTTCTACATTTCCAAGACC | CCCGGTAGGCGTCAGGTAT |
| Mus musculus CD146 | CCCAAACTGGTGTGCGTCTT | GGAAAATCAGTATCTGCCTCTCC |
| Mus musculus PDGFR-B | CGGATGTCACTGAGACGACAA | AGTAAGTATCGGAGTCCACTTCC |
| Mus musculus ERA | CCTCCCGCCTTCTACAGGT | CACACGGCACAGTAGCGAG |
| Mus musculus ERB | CTGTGCCTCTTCTCACAAGGA | TGCTCCAAGGGTAGGATGGAC |
| Mus musculus Foxa2 | CCCTACGCCAACATGAACTCG | GTTCTGCCGGTAGAAAGGGA |
| Mus musculus Hand1 | GGCAGCTACGCACATCATCA | GCATCGGGACCATAGGCAG |
| Mus musculus Hoxa10 | CCGCGAACTCCTTTTTGGTC | GCTTCATTACGCTTGCTGCC |
| Mus musculus PR-A | CTACTCGCTGTGCCTTACCA | CTTTGACTCCTCAGTCCTTCCA |
| Mus musculus PR-B | GCGCTTCTACCAACTCACATA | GGCAGCAATAACTTCAGACATCA |
