# Supplementary figures and images for: Differentiating mouse embryonic stem cells express markers of human endometrium
Source: Reprod Biol Endocrinol. 2017 Jul 17;15:52. doi: 10.1186/s12958-017-0273-2 (PMC5514487; doi:10.1186/s12958-017-0273-2)

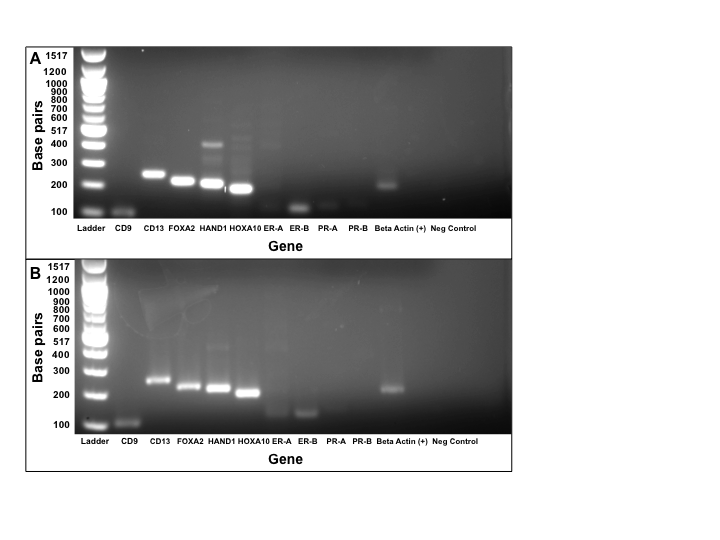

Supplement: Supplementary file 2 — Gene expression in differentiating EBs in Week 1 and Week 2. RT-PCR demonstrates expression of CD9, CD13, Foxa2, Hand1, Hoxa10, ER-A and -B, and PR-A and -B in Week 1 (A) and 2 (B) of EB differentiation. The ladder is indicated on the left and positive and negative controls are shown in far right columns. (TIFF 1521 kb) [file 12958_2017_273_MOESM2_ESM.tiff]

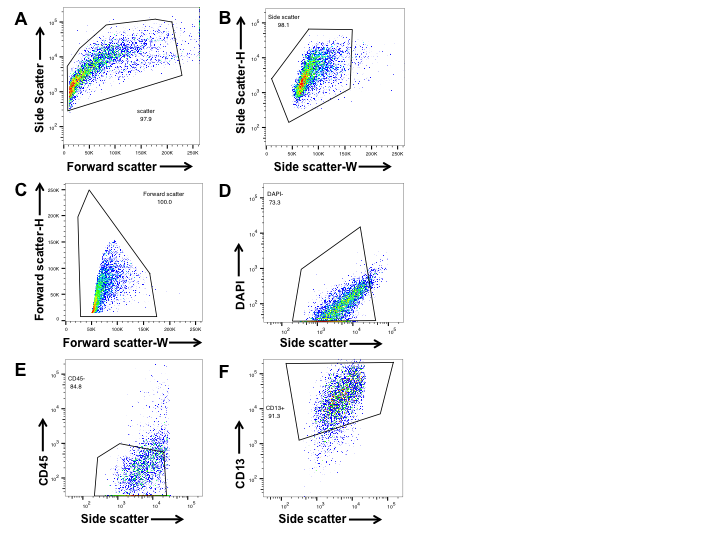

Supplement: Supplementary file 3 — Schema of complete gating of FACS-sorted CD13+ cells in EB media. Total cells were first gated to exclude debris with forward and side scatter plots (A-C). Next, viable cells were negatively selected with DAPI (D). CD45- cells were selected (E), followed by CD13+ cells (F). Graph legends indicate percent of parent population. (TIFF 1521 kb) [file 12958_2017_273_MOESM3_ESM.tiff]
